# Mind the Gap—A Perspective on Strategies for Protecting against Bacterial Infections during the Period from Infection to Eradication

**DOI:** 10.3390/microorganisms11071701

**Published:** 2023-06-29

**Authors:** Yoav Gal, Hadar Marcus, Emanuelle Mamroud, Ronit Aloni-Grinstein

**Affiliations:** 1 Department of Biochemistry and Molecular Genetics, Israel Institute for Biological Research, Ness Ziona 74100, Israel; yoavg@iibr.gov.il (Y.G.); emmym@iibr.gov.il (E.M.); 2 Department of Biotechnology, Israel Institute for Biological Research, Ness Ziona 74100, Israel; hadarm@iibr.gov.il

**Keywords:** innate immunity, therapeutic vaccines, nutritional immunity, neutrophil activation, TLR ligands, adjuvants, antimicrobial resistance

## Abstract

The emergence of antibiotic-resistant bacteria is a pressing public health concern, highlighting the need for alternative approaches to control bacterial infections. Promising approaches include the development of therapeutic vaccines and the utilization of innate immune activation techniques, which may prove useful in conjunction with antibiotics, as well as other antibacterial modalities. However, innate activation should be fast and self- or actively- contained to prevent detrimental consequences. TLR ligand adjuvants are effective at rapidly activating, within minutes to hours, the innate immune system by inducing cytokine production and other signaling molecules that bolster the host’s immune response. Neutrophils serve as the first line of defense against invading pathogens by capturing and destroying them through various mechanisms, such as phagocytosis, intracellular degradation, and the formation of NETs. Nutritional immunity is another host defense mechanism that limits the availability of essential metals, such as iron, from invading bacterial pathogens. Thus, iron starvation has been proposed as a potential antibacterial strategy. In this review, we focus on approaches that have the potential to enhance rapid and precise antibacterial responses, bridging the gap between the onset of infection and the elimination of bacteria, hence limiting the infection by antibiotic-resistant bacteria.

## 1. Introduction

### Novel Strategies for Combating AMR Bacterial Infections

Antimicrobial resistance (AMR) is a “silent pandemic” that, with time, gains more public health concerns, both at the community level and more severely in hospital settings. In 2019, AMR was estimated to be associated with 4.95 million deaths worldwide [1]. By 2050, the death toll due to AMR is predicted to be ten million per year [2]. High AMR rates may also influence other medical procedures, such as surgeries, organ transplantations, kidney diseases, and cancer treatments, where bacteria presence may create a serious medical condition. Therefore, it is urgent to seek effective options that will reduce the daily use of antibiotics, allow for tight stewardship of antibiotic use to decrease AMR formation, and increase the antimicrobial efficiency of antibiotics.

Herein, in this perspective review, we challenge alternative antimicrobial strategies. Although these alternatives are in their preclinical stages, may have side effects, are too expensive for daily use, and/or not even considered currently as an optional treatment, we believe that the increasing rates of AMR would inevitably force the development and improvement of these approaches. Indeed, optional mimicry or synthetic alternatives to naturally occurring antibacterial mechanisms/molecules are being developed. However, more research is needed.

One potential alternative approach for both prophylaxis and therapy of bacterial infections may be vaccines. Vaccines directed against *Streptococcus pneumoniae*, *Haemophilus influenzae* type b, *Mycobacterium tuberculosis*, and *Salmonella enterica* serotype Typhi are licensed, and many others are at various stages of clinical and pre-clinical development [3]. The licensed vaccines are based on the classic mechanism of adaptive immunity, where a bacterial antigen is introduced to the immune system, which generates antibodies and memory B cells that are specific to the introduced antigen/s. An alternative solution to traditional, highly selective vaccines may be non-specific vaccines, which may initiate rapid responses post challenge through the activation of the innate system. The BCG vaccine is believed to induce innate, as well as T-cell responses to several pathogens that do not possess a common antigen with BCG [4]. Beyond vaccines, a promising strategy for combating bacterial infections is to elicit rapid responses, within hours to days that provide quick and effective post-exposure protection when concomitantly administered with traditional antibiotic agents or other antibacterial modalities. Such protection may be provided by several mechanisms, which are based mostly on innate immune activation, which enable the closure of the gap between the time of infection and the onset of the antimicrobial effect.

In this review, we focus on the latest progress in the field, particularly on three potential antibacterial modalities: post-exposure administration of adjuvants and induction of protective immunity by therapeutic vaccines, neutrophil activation, and nutritional immunity (iron chelation), all of which induce rapid and efficient activation of the innate immune response.

## 2. Strategies for Facilitating Quick Antibacterial Responses

### 2.1. Adjuvants

Adjuvants are substances that can enhance the immune response to vaccine components, potentially strengthening both innate and adaptive immune responses. By activating cellular and humoral responses, adjuvants can reduce the amount of an antigen required for efficient vaccination, improving immunogenicity in populations that typically respond poorly, such as the elderly or immunocompromised. Furthermore, adjuvants, such as AS03 (GSK) [5,6,7,8,9], MF59 (Novartis Vaccines and Diagnostics) [5,6,7,8,9,10,11,12,13], Matrix-M (Novavax) [14,15], and monophosphoryl lipid A (MPL) (GlaxoSmithKline), have encouraging safety profiles.

Traditional vaccine adjuvants, including various Toll-like receptor (TLR) ligands, can activate the innate immune system within minutes to hours, resulting in the production of cytokines and other signaling molecules that further enhance the immune response [16]. This TLR-mediated rapid immune activation, which can last for several days, is part of the natural host’s first line of defense, which is critical for preventing the further spread of the infection. Moreover, it provides the necessary groundwork and time for subsequent activation of the adaptive immune response.

Adjuvants, such as AS02, AS03, AS04, MF59, MPL, and Matrix-M, are used in some vaccines to enhance the immune response against viral and bacterial infections. AS03 and MPL activate Toll-like receptor 4 (TLR4), while MF59 activates TLR4 + TLR8, and Matrix-M activates TLR4 + TLR3. These adjuvants have been shown to increase the activation and function of various immune cells, including dendritic cells, neutrophils, natural killer (NK), and T and B cells [17,18,19,20,21,22,23,24].

Moreover, these adjuvants stimulate the production of cytokines, such as interferon γ (IFN-γ), interleukin 1α (IL-1α), IL-1β, IL-6, tumor necrosis factor (TNF)-α, IL-8, and IL-12. These cytokines activate and enhance the ability of immune cells, such as neutrophils, macrophages, dendritic cells, and NK cells, to engulf and destroy invading viruses and bacteria [19,20,21,22,23,24,25].

Additionally, these adjuvants stimulate the production of other components, such as chemokines, that are crucial for the immune response. For example, AS03 activates the production of the chemokines C-C motif CCL2, CCL3, and CCL5, which attract immune cells to the vaccine site, further enhancing the host’s immune response [21,23,26]. Thus, it could be that these adjuvants may also be used outside the context of a vaccine, namely, as immune stimulants to be concomitantly administered with post-exposure antibacterial treatments.

Indeed, although most clinically approved adjuvants were developed as anti-viral tools, some have also been found effective against bacteria, such as *Streptococcus pneumoniae*, *Haemophilus influenzae* type b [27], and *Mycobacterium tuberculosis* [28]. In animal studies, MF59 has been found to enhance the immunogenicity and protective immunity against *Acinetobacter baumannii* [29], while a combination of a recombinant protein and MPL was found to be immunogenic against *Neisseria meningitis* [30].

To conclude, promising new adjuvants might contribute to the closure of the gap by efficient immune responses for bacterial clearance when administered immediately post exposure, even in the absence of a specific antigen. To note, some adjuvants may only be efficient in the presence of an additional antibacterial agent. In our opinion, it is important to keep tracking progress in these fields, including drug delivery systems (nanoparticle-based adjuvants, virus-like particles), novel TLR agonists, and combinational adjuvant treatments.

### 2.2. Rapid Induction of Protective Immunity by Therapeutic Vaccines

Most vaccines, which are prophylactic in nature, focus on enhancing the adaptive immune response to generate a high and fast antimicrobial effect when encountered in the future with the pathogen. However, vaccines may be also used therapeutically against viruses and bacteria if activation of the innate immune system takes place fast enough to close the gap until the adaptive immune response joins the efforts to eradicate the pathogen.

Studies from our group have shown that the *Yersinia pestis* EV76 live vaccine protected mice against an immediate subcutaneous lethal challenge. Strikingly, immunization two days prior to pulmonary infection also provided protection [31]. Subcutaneous immunization with a *Y. pestis* strain (Kim53ΔJ+P) that over-expresses *Y. enterocolitica* YopP also elicited a fast and effective protective immune response in models of bubonic, pneumonic, and septicemic plague through the induction of a prompt protective innate immune response that was interferon-γ dependent. Moreover, cross-protection to other bacterial pathogens, such as the enteropathogen *Y. enterocolitica* that causes Yersinosis and *Francisella tularensis*, the causative agent of Tularemia, was attained [32]. Immunization, pre (adjacent) or, post-exposure to a lethal *Y. pestis* infection, with another anti-*Y. pestis* vaccine, based on the F1 -recombinant protein adsorbed on alum hydroxide, also provided rapid protection in the bubonic plague mice model through anti-F1 IgM and IgG antibodies that developed within a few days post-vaccination. This line of protection was attributed to the activation of innate-like B cell subsets [33,34]. In a following study, it was shown that effective protection against subsequent lethal intranasal exposure to a fully virulent *Y. pestis* strain is obtained within a week following immunization with F1 adsorbed on alum hydroxide and that the addition of the LcrV antigen reduced the time to generate protective immunity to four to five days after vaccination [35]. It is intriguing to believe that therapeutic vaccines could be an add-on post-exposure treatment if administered with antibiotics or other antimicrobial treatments.

In a recent study, intranasal immunization with a single dose of inactivated whole-cell *Acinetobacter baumannii* vaccination provided protection from a lethal dose of *Acinetobacter baumannii* as early as two days after immunization [36]. Cross protection was also seen in *Klebsiella pneumonia* and *Pseudomonas aeruginosa* pneumonia models. Protection of the immunized mice was correlated with elevated levels of IL-6 and TNF-α, which decreased by day five. However, rapid recall responses to infection were observed after a challenge on day seven post-vaccination. TNF-α secretion and chemokine production was noticed two hours post-challenge followed by neutrophil infiltration, as early as 4 h post infection. This response was attributed to immunization-trained alveolar macrophages, posing upregulated TLR4 expression, which mediated the rapid protection through enhanced TNF-α production [36].

To conclude, we believe that our work on *Y. pestis* vaccines offers a proof-of-concept to this strategy, which is applicable within days of administration, and this line of therapy should be perused and even examined as a post-exposure countermeasure.

### 2.3. Neutrophil Activation

Neutrophils play a crucial role as the first line of defense against invading pathogens, utilizing phagocytosis and intracellular degradation, as well as the release of granules, reactive oxygen species (ROS), and neutrophil extracellular traps to capture and destroy them [37,38]. In contrast to macrophages, which have a long half-life of weeks [39,40] or even years [41], neutrophils have a significantly shorter half-life (hours-days) [42,43], making them more suitable for promptly controlled activation against bacteria. However, although critical for bacterial killing, neutrophils may induce collateral damage and tissue injury by amplifying inflammation [44,45]. Therefore, when inducing protection via an innate neutrophil-mediated immune response, there should always be a delicate balance between prompt bactericidal effect (especially in the case of bacteria that silence primary neutrophil effects, as in granule release inhibition following *Yersinia pestis* infection [46] or recruitment inhibition following *Staphylococcus aureus* infection [47]) and timely resolution of neutrophils’ potentially damaging effect. Strategies for neutrophil activation and resolution are discussed below.

#### 2.3.1. Neutrophil Activation via Formylated Peptides and Formylated Peptide Receptors’ (FPRs) Modulators

Formylated peptides are key pathogen-associated molecules that activate innate response by binding to formyl peptide receptors (FPRs) and stimulating chemotaxis and neutrophil-mediated phagocytosis of bacteria [48,49]. FPR1 and FPR2 are crucial for bacterial immune defense, as FPR1- and FPR2-deficient mice are characterized by increased susceptibility to *Listeria monocytogenes* infection and meningitis caused by *Streptococcus pneumoniae*, in comparison to wild-type mice [50,51]. It was recently demonstrated, in vitro, that FPR2 activation enhances neutrophil-induced phagocytosis and bacterial killing of community-acquired methicillin-resistant *Staphylococcus aureus* [52].

FPR modulators could offer opportunities to develop novel antibacterial therapeutics; this approach may be tricky, since FPRs are highly expressed not only in phagocytes, but also in non-hematopoietic cells, such as epithelial- and endothelial-cells [53]. In addition, formylated peptides can activate leukocytes at picomolar concentrations [54], consequently leading to excess leukocyte activation, tissue damage, organ dysfunction, and mortality. In this regard, compounds modulating FPRs’ activity have been examined for their potential to counteract pathological conditions, such as inflammatory lung diseases, ischemia-reperfusion injury, and sepsis [55]. However, although the main goal of the research and the development of FPR modulators (agonists and antagonists), including progress in rational drug design, is combating inflammation and other injuries [56], these modulators, in particular small molecules under clinical evaluation, may be utilized, in our opinion, to induce a better antibacterial effects via neutrophil activation, and, in addition, small molecule receptor modulators may be utilized for safety purposes, namely, to actively counteract over-activation of FPRs after completion of the bactericidal effect. These small molecules may be selective to a certain FPR or to a receptor subtype/combination of FPRs [57]. For example, in a recent study, it was shown that the small molecule RE-04-001 activates human neutrophils, and this agonist is specifically recognized by FPR1, owing to a functional selective response via activation of the ROS, generating NADPH-oxidase in neutrophils (rather that neutrophil chemotaxis) [58]. A non-peptide compound, Quin-C1, a FPR2 agonist characterized two decades ago, was found to be a selective inducer of neutrophil chemotaxis, rather than a ROS generator [59].

Other means to amplify neutrophil-derived ROS formation were also suggested [60], and, in our opinion, they may be used to combat bacteria. However, this subject is beyond the scope of this review.

A more elegant strategy suggested recently for using formylated peptides for combating bacteria is via antibiotic-chemoattractant conjugates, consisting, for example, of a formylated-peptide covalently linked to an antibiotic, such as vancomycin. A formylated-peptide/vancomycin conjugate demonstrated improved neutrophil recruitment and enhanced bactericidal effects against *S. aureus*. This immunotherapeutic antibiotic was suggested to overcome immune evasion via the dual effect of bacterial eradication and fine-tuned neutrophil recruitment [61].

#### 2.3.2. Neutrophil Extracellular Traps (NETs) Formation and NETs Mimicry

A crucial mechanism of neutrophil rapid defense is NET formation (NETosis), an immediate immune response taking place following the microbial invasion. NETs are composed of extracellular DNA, chromatin, proteolytic enzymes, and antimicrobial peptides, forming extracellular fibers that bind bacteria, enabling both pathogen inactivation and spreading prevention [62]. In addition, NETosis may augment macrophage-induced killing of bacteria, namely, neutrophils are activated in concert with macrophages to effectively eradicate bacterial pathogens [63]. Although NETosis may be associated with severe tissue damage, as well as other pathophysiological states [64], it may very well be that spatially and temporally controlled NETosis promotes bacterial killing while reducing the pathological consequence. In this regard, it was recently demonstrated that memantine, a drug used to treat Alzheimer’s disease, exerts bactericidal effects against an MDR *Escherichia coli* via enhancing bacterial-induced NETosis in vivo [65]. It was also demonstrated that some β-Lactams modulate NETosis in activated neutrophils in vitro, via a non-antibacterial effect, namely, the activation of the PKC/Akt/mTOR signal pathway, thus exerting a dual effect (antibacterial and immune modulation) [66], enabling their administration for the treatment of β-Lactam-resistant strains in combination with relevant antibiotics for an improved bactericidal effect.

The detrimental NETs-induced effects may be actively reduced, following their active induction, by dampening NETosis immediately following bacterial clearance, via resolvin administration. Resolution of acute inflammatory response is now widely recognized as an active process, via the biosynthesis of endogenous pro-resolving lipid mediators acting on innate system components [67]. Thirteen-series resolvins are potent agonists of pro-resolving phagocyte functions accelerating resolution, i.e., via controlling inflammation. It was recently demonstrated that 13-series resolvins reduce NETosis and stimulate macrophage clearance of NETs [67], and, thus, these molecules may be used to actively and timely control NETosis. An alternative approach for spatially and temporally “breaking the NETs”, at least in the case of a respiratory bacterial infection, may be the administration of clinically approved drugs, such as pulmozyme, that has the potential to reduce NETs and aid recovery, which was demonstrated in COVID-19 patients [68], thus hopefully may be applicable also for bacterial infections.

In addition to memantine mentioned above, which induced NETosis in vivo, other clinically approved drugs were shown to increase NET formation in vitro, including hydralazine and procainamide [69]. Importantly, the clinical concern of NET formation demonstrated for these drugs is their potential to induce autoimmunity. However, this phenomenon usually develops only after months and, more commonly, years of treatment [70]. Thus, a short-duration treatment may improve the antibacterial effect via NETs formation. Likewise, NETs were developed in mice following melatonin treatment, resulting in anti-bacterial activity during polymicrobial infection [71]. In line with that, we suggest conducting high throughput in vitro screening of clinically approved drugs for their potential to induce NETs, offering novel antibacterial candidates, particularly for drugs with reasonable safety profiles (as standalone antibacterial, or in combination with antibiotics or other antibacterial agents). “Synthetic NETs” are also being designed to combat antibiotic-resistant organisms or to be used as an adjunct to conventional antibacterial treatment. In this respect, a minimalistic NET-like synthetic structure, termed “microwebs”, demonstrated direct in vitro antibacterial effects, as well as a synergistic effect with colistin [72]. Other NET-like structures composed of DNA nanogels and ZnO nanoparticles suppressed *E. coli* from entering circulation in septic mice while prolonging their survival, possibly through an anti-inflammatory effect, in contrast to a direct bactericidal effect [73]. More recently, it was reported that self-assembled antimicrobial peptide nanonets are capable of in vitro and in vivo antibacterial activity, including against antibiotic-resistant bacteria, thus enabling spatiotemporal control over microbial killing. The peptide nanonets formation was induced by the lipopolysaccharide (LPS) of Gram-negative bacteria and the lipoteichoic acid (LTA) of Gram-positive ones and were shown to be stable against proteolytic degradation. Most importantly, these peptides were shown to be safe, as they lacked toxicity up to 24 h post administration [74]. Others have reported on an in vivo supramolecular assembly system that imitates a nanofibers network that efficiently trapped methicillin-resistant *staphylococcus epidermidis* (MRSE) bacteria, hence providing antibacterial activity and the prevention of extensive dissemination [75]. All in all, the progress in this field is intriguing and should be closely monitored.

To conclude, the manipulation of neutrophils is a suitable approach for promptly controlled activation against antibiotic-resistant bacteria. However, there should always be a delicate balance between prompt bactericidal effect and timely resolution of neutrophils’ potentially damaging effect. Strategies for neutrophil activation and resolution, such as the use of NETosis and formylated peptides, offer, in our opinion, promising opportunities for developing novel anti-bacterial therapeutics.

### 2.4. Nutritional Immunity

Various metals, such as iron, manganese, zinc, and copper, are essential for the cellular functions of bacterial pathogens. Therefore, it is not surprising that the host undergoes significant changes in response to bacterial infections to regulate metal availability as a means of defense. The host’s innate immune response, which involves the withholding of metal nutrients to prevent bacterial growth, is defined as nutritional immunity [76,77]. For comprehensive reviews on nutritional immunity, please see [77,78]. In this review, we will focus on iron starvation.

#### 2.4.1. Iron Starvation

Bacteria have highly efficient mechanisms to obtain iron from their surroundings, such as receptors that bind host iron proteins, expression of high-affinity iron transporters, and the use of low-molecular-weight-compounds with high iron-binding affinities, such as siderophores and haemophores [79]. As a countermeasure, the host has devised many strategies to sequester iron from invading pathogens.

Hepcidin (hep-liver, cidin-antibacterial), a 25 amino acid peptide hormone, which regulates iron homeostasis, is secreted by the liver upon an inflammatory stimulus [80,81]. Hepcidin was first noticed in a patient with a systemic infection, with more than a 100- fold increase in this peptide found in his urine [82]. Inflammatory stimuli, particularly IL-6, upregulate Jak2/STAT3 signaling-induced hepatic hepcidin production. Thereafter, hepcidin signals for the internalization and degradation of the iron exporter, ferroportin, leading to the inhibition of iron transfer from iron storage in enterocytes and macrophages into blood circulation. Elevated levels of hepcidin have been demonstrated in mice following *Salmonella* [83], *Pseudomonas aeruginosa* [84], group A Streptococcus [84], *Vibrio vulnificus* [85], and *Candida albicans* or *Influenza A* virus [86] infections. Similarly, transporters, such as natural resistance-associated macrophage protein 1 (NRAMP1), which are localized in the lysosomes and phagosomes of monocytes, macrophages, and T lymphocytes, are also recruited by the host to withhold iron from pathogens [87].

Hemopexin (HPX) is a plasma protein with a high binding affinity to heme, and its synthesis is induced following inflammation [88]. Our research group has demonstrated that vaccination with a live attenuated *Yersinia pestis* strain provided immediate protection against a lethal challenge in a mouse model of bubonic plague, which involves the rapid induction of HPX expression occurring simultaneously with vaccination and delaying the progression of the disease until the development of protective antibodies [31]. Consistent with this finding, exogenous HPX treatment has been shown to modulate the severity of *P. aeruginosa* pneumonia in mice [89]. Other studies have shown that IL-22 induces HPX expression, which could protect mice against the enteric pathogen *C. rodentium* and the commensal *E. coli* [90].

Recently, another mechanism of iron depletion from the serum has been described. Upon infection, macrophages induce the formation and release of extracellular vesicles (EV), containing iron protein, such as transferrin receptor (TfR), LDL-related 64 protein 1 (CD91), and hemoglobin-haptoglobin receptor (CD163) [91,92]. These EVs may engulf serum iron proteins, including iron-loaded transferrin, leading to low serum iron levels [91,92]. In a *Salmonella Typhimurium* mouse model, the concentration of EVs in serum increased at 3 h after infection and continued to increase within 24 h, indicating that EVs are a means for prompt iron sequestration and resistance to infection [92].

Neutrophils (discussed in Section 2.3) also play a role in nutritional immunity by secreting: lactoferrin, an iron-binding glycoprotein found in neutrophil secondary granules and decorating neutrophil extracellular traps (NETs) [93]; calprotectin, a heterodimer of the subunits S100A8 and S100A9 that exerts its bacteriostatic activity through the sequestration of zinc, manganese, and iron [94,95]; and lipocalin-2 (Lcn2) (also known as siderocalin, neutrophil-associated gelatinase lipocalin), which sequesters bacterial siderophores [96,97,98,99].

#### 2.4.2. Therapeutic Strategies of Nutritional Immunity

Iron chelators have been previously suggested as a potential adjunct therapy in murine models, preferably together with conventional antibiotics [100,101,102,103,104]. However, their toxicity and potential to deliver iron to the bacteria limit their use [105]. To overcome these limitations, exogenous administration of hepcidin has been suggested as an alternative to the body’s natural hypoferremic pathways. This therapeutic modality is expected to be less toxic and more efficient. Alternately, endogenous hepcidin induction may be achieved by catecholamines (such as norepinephrine and dopamine) administration [105]. Hepcidin can be used in conjunction with standard therapy in patients with iron overload, suffering from deadly infections of siderophilic Gram-negative bacilli, such as *Yersinia enterocolitica*, *Vibrio vulnificus,* and *Klebsiella pneumoniae*.

Another potential strategy is the use of a modified Lcn2 with an increased half-life, tissue penetration, and target specificity [106]. Lcn2 has been shown to play a role in various health conditions, and more than 70 clinical trials are ongoing to investigate its therapeutic potential [107]. In addition, the administration of EVs bearing TfR-, CD163-, and CD91- (as discussed in Section 2.4.1) can prevent iron from bacteria.

To conclude, therapeutic iron starvation monitored by repurposing drugs, such as catecholamines or endogenous/synthetic biological molecules that were shown to be involved in iron depletion, such as hepcidin and Lcn2, or the use of TfR-, CD163-, and CD91-bearing EVs, may be also administered as standalone treatments or complement other antibacterial strategies.

## 3. Conclusions

The growing threat of AMR should place, in the front, studies aiming at developing future antimicrobial means. There are various non-antibiotic-countermeasure approaches against AMR bacteria, such as monoclonal antibodies and phage treatments, immune modulation by clinically approved drugs (‘antibiotic adjuvants’), and more [108]. Clinical proofs-of-concept were demonstrated for all of these. In this perspective review, we aim to highlight and challenge a scientific approach that intertwines various strategies to enhance antibacterial effects via rapid, accurate, and effective stimulation of particular components of the innate system. Not only these approaches should be rapid and efficient, but also some of them should be self- or actively contained to avoid adverse effects on the host due to overstimulation of neutrophils or prolonged nutritional depletion. Note that the antibacterial strategies presented in this review are in their earliest stages of research and development and may possess hurdles, such as side effects and delivery issues. Yet, accumulating data from various fields of studies may offer solutions that may pave the way to future progress (Table 1).

In light of the progress in drug delivery, synthetic biology, and drug development, some of the potential safety issues to the suggested approaches may be managed. Moreover, studies in cancer therapy have pointed to the vast potential of therapeutic adjuvants as immune stimulators and/or therapeutic vaccines. The continuous progress in that field may have a great impact, as re-purposed treatments, on antibacterial treatments, in particular, in cases of AMR microbial infections. We believe it is worthwhile keeping abreast of the progress in these potential treatment strategies.

## Figures and Tables

**Table 1:** Potential approaches for protecting against bacterial Infections during the period from infection to eradication.

Antibacterial Approach	Potential Antibacterial Moiety	Response Onset	Mechanism	Remarks
TLR ligand adjuvants	AS03 (TLR4)	Minutes-hours	Enhancement of immune response via cytokine, chemokines, and signaling molecule production	Should be co-administered with other antibacterial agent/s
	MF59 (TLR4, TLR8)			
	Matrix-M (TLR3, TLR4)			
	MPL (TLR4)			
Therapeutic vaccines	*Y. pestis* EV76	Hours-days	Rapid induction of protective immune response via cytokine and chemokine production	
	*Y. pestis* Kim53ΔJ+P			IFNγ-dependent response Protective against *F. Tularensis* and *Y. enterocolitica*
	Polymeric F1 protein+Alum			Innate-like B cell subset activation
	*Acinetobacter baumannii*			*K. pneumoniae* and *P. aeruginosa* cross protection
Neutrophil activation	Formylated peptides	Minutes	Stimulation of neutrophil chemotaxis and phagocytosis via FPR binding	Picomolar concentrations activation
	Antibiotic-chemoattractant conjugates	Minutes	Improving recruitment, engulfment, and killing of bacteria by neutrophils	
	NETosis	Minutes–hours	Bacteria trapping and killing	May be harmful if not controlled. Should be actively terminated (i.e., via resolvins) or induced with drugs of well known safety profiles (i.e., memantine).
	Synthetic NETs	Minutes–hours	Bacteria trapping and killing	Some are useful in the presence of antibacterial agents.
Nutritional immunity	Hepcidin	Hours	Iron starvation (internalization and degradation of ferroportin)	May be induced by catecholamines
	Hemopexin		Iron starvation (Heme binding)	
	extracellular vesicles (EV) formation		Iron starvation (engulfing serum iron proteins, leading to low serum iron levels)	The EV contain TfR, LDL-related 64 protein 1 (CD91), and hemoglobin-haptoglobin receptor (CD163)
	Lactoferrin, calprotectin and LCN2		Neutrophil-derived sequestration of iron and bacterial siderophores	Neutrophil-derived proteins

## References

[1] Antimicrobial Resistance C. (2022). Global burden of bacterial antimicrobial resistance in 2019: A systematic analysis. Lancet.

[2] O’Neill J. (2016). Tackling Drug-Resistant Infections Globally: Final Report and Recommendations.

[3] Frost I., Sati H., Garcia-Vello P., Hasso-Agopsowicz M., Lienhardt C., Gigante V., Beyer P. (2022). The role of bacterial vaccines in the fight against antimicrobial resistance: An analysis of the preclinical and clinical development pipeline. Lancet Microbe.

[4] Pollard A.J., Bijker E.M. (2021). A guide to vaccinology: From basic principles to new developments. Nat. Rev. Immunol..

[5] Fox C.B., Haensler J. (2013). An update on safety and immunogenicity of vaccines containing emulsion-based adjuvants. Expert Rev. Vaccines.

[6] Tetsutani K., Ishii K.J. (2012). Adjuvants in influenza vaccines. Vaccine.

[7] Yin J.K., Khandaker G., Rashid H., Heron L., Ridda I., Booy R. (2011). Immunogenicity and safety of pandemic influenza A (H1N1) 2009 vaccine: Systematic review and meta-analysis. Influenza Other Respir. Viruses.

[8] Durando P., Iudici R., Alicino C., Alberti M., de Florentis D., Ansaldi F., Icardi G. (2011). Adjuvants and alternative routes of administration towards the development of the ideal influenza vaccine. Hum. Vaccines.

[9] Atmar R.L., Keitel W.A. (2009). Adjuvants for pandemic influenza vaccines. Curr. Top. Microbiol. Immunol..

[10] Gray G.E., Bekker L.G., Laher F., Malahleha M., Allen M., Moodie Z., Grunenberg N., Huang Y., Grove D., Prigmore B. (2021). Vaccine Efficacy of ALVAC-HIV and Bivalent Subtype C gp120-MF59 in Adults. N. Engl. J. Med..

[11] Chappell K.J., Mordant F.L., Li Z., Wijesundara D.K., Ellenberg P., Lackenby J.A., Cheung S.T.M., Modhiran N., Avumegah M.S., Henderson C.L. (2021). Safety and immunogenicity of an MF59-adjuvanted spike glycoprotein-clamp vaccine for SARS-CoV-2: A randomised, double-blind, placebo-controlled, phase 1 trial. Lancet Infect. Dis..

[12] Baraniak I., Kern F., Holenya P., Griffiths P., Reeves M. (2019). Original Antigenic Sin Shapes the Immunological Repertoire Evoked by Human Cytomegalovirus Glycoprotein B/MF59 Vaccine in Seropositive Recipients. J. Infect. Dis..

[13] Baraniak I., Kropff B., McLean G.R., Pichon S., Piras-Douce F., Milne R.S.B., Smith C., Mach M., Griffiths P.D., Reeves M.B. (2018). Epitope-Specific Humoral Responses to Human Cytomegalovirus Glycoprotein-B Vaccine with MF59: Anti-AD2 Levels Correlate With Protection From Viremia. J. Infect. Dis..

[14] Heath P.T., Galiza E.P., Baxter D.N., Boffito M., Browne D., Burns F., Chadwick D.R., Clark R., Cosgrove C., Galloway J. (2021). Safety and Efficacy of NVX-CoV2373 COVID-19 Vaccine. N. Engl. J. Med..

[15] Zhou F., Hansen L., Pedersen G., Grodeland G., Cox R. (2021). Matrix M Adjuvanted H5N1 Vaccine Elicits Broadly Neutralizing Antibodies and Neuraminidase Inhibiting Antibodies in Humans That Correlate with In Vivo Protection. Front. Immunol..

[16] Calabro S., Tortoli M., Baudner B.C., Pacitto A., Cortese M., O’Hagan D.T., De Gregorio E., Seubert A., Wack A. (2011). Vaccine adjuvants alum and MF59 induce rapid recruitment of neutrophils and monocytes that participate in antigen transport to draining lymph nodes. Vaccine.

[17] Ko E.-J., Lee Y., Lee Y.-T., Kim Y.-J., Kim K.-H., Kang S.-M. (2018). MPL and CpG combination adjuvants promote homologous and heterosubtypic cross protection of inactivated split influenza virus vaccine. Antivir. Res..

[18] Martin M., Michalek S.M., Katz J. (2003). Role of innate immune factors in the adjuvant activity of monophosphoryl lipid A. Infect. Immun..

[19] Howard L.M., Hoek K.L., Goll J.B., Samir P., Galassie A., Allos T.M., Niu X., Gordy L.E., Creech C.B., Prasad N. (2017). Cell-Based Systems Biology Analysis of Human AS03-Adjuvanted H5N1 Avian Influenza Vaccine Responses: A Phase I Randomized Controlled Trial. PLoS ONE.

[20] Caproni E., Tritto E., Cortese M., Muzzi A., Mosca F., Monaci E., Baudner B., Seubert A., De Gregorio E. (2012). MF59 and Pam3CSK4 boost adaptive responses to influenza subunit vaccine through an IFN type I-independent mechanism of action. J. Immunol..

[21] Seubert A., Monaci E., Pizza M., O’Hagan D.T., Wack A. (2008). The adjuvants aluminum hydroxide and MF59 induce monocyte and granulocyte chemoattractants and enhance monocyte differentiation toward dendritic cells. J. Immunol..

[22] Morel S., Didierlaurent A., Bourguignon P., Delhaye S., Baras B., Jacob V., Planty C., Elouahabi A., Harvengt P., Carlsen H. (2011). Adjuvant System AS03 containing alpha-tocopherol modulates innate immune response and leads to improved adaptive immunity. Vaccine.

[23] Hwang S.J., Chang S.C., Yu C.J., Chan Y.J., Chen T.J., Hsieh S.L., Lai H.Y., Lin M.H., Liu J.Y., Ong G. (2011). Immunogenicity and safety of an AS03(A)-adjuvanted H5N1 influenza vaccine in a Taiwanese population. J. Formos. Med. Assoc..

[24] Magnusson S.E., Reimer J.M., Karlsson K.H., Lilja L., Bengtsson K.L., Stertman L. (2013). Immune enhancing properties of the novel Matrix-M adjuvant leads to potentiated immune responses to an influenza vaccine in mice. Vaccine.

[25] Dooling K.L., Guo A., Patel M., Lee G.M., Moore K., Belongia E.A., Harpaz R. (2018). Recommendations of the Advisory Committee on Immunization Practices for Use of Herpes Zoster Vaccines. MMWR Morb. Mortal. Wkly. Rep..

[26] Ko E.J., Kang S.M. (2018). Immunology and efficacy of MF59-adjuvanted vaccines. Hum. Vaccin. Immunother..

[27] Berglund J., Vink P., Tavares Da Silva F., Lestrate P., Boutriau D. (2014). Safety, immunogenicity, and antibody persistence following an investigational Streptococcus pneumoniae and Haemophilus influenzae triple-protein vaccine in a phase 1 randomized controlled study in healthy adults. Clin. Vaccine Immunol..

[28] Tait D.R., Hatherill M., Van Der Meeren O., Ginsberg A.M., Van Brakel E., Salaun B., Scriba T.J., Akite E.J., Ayles H.M., Bollaerts A. (2019). Final Analysis of a Trial of M72/AS01(E) Vaccine to Prevent Tuberculosis. N. Engl. J. Med..

[29] Yang A.Q., Yang H.Y., Guo S.J., Xie Y.E. (2019). MF59 adjuvant enhances the immunogenicity and protective immunity of the OmpK/Omp22 fusion protein from Acineterbacter baumannii through intratracheal inoculation in mice. Scand. J. Immunol..

[30] Kortekaas J., Muller S.A., Ringler P., Gregorini M., Weynants V.E., Rutten L., Bos M.P., Tommassen J. (2006). Immunogenicity and structural characterisation of an in vitro folded meningococcal siderophore receptor (FrpB, FetA). Microbes Infect..

[31] Zauberman A., Vagima Y., Tidhar A., Aftalion M., Gur D., Rotem S., Chitlaru T., Levy Y., Mamroud E. (2017). Host Iron Nutritional Immunity Induced by a Live Yersinia pestis Vaccine Strain Is Associated with Immediate Protection against Plague. Front. Cell. Infect. Microbiol..

[32] Zauberman A., Flashner Y., Levy Y., Vagima Y., Tidhar A., Cohen O., Bar-Haim E., Gur D., Aftalion M., Halperin G. (2013). YopP-expressing variant of Y. pestis activates a potent innate immune response affording cross-protection against yersiniosis and tularemia [corrected]. PLoS ONE.

[33] Levy Y., Flashner Y., Tidhar A., Zauberman A., Aftalion M., Lazar S., Gur D., Shafferman A., Mamroud E. (2011). T cells play an essential role in anti-F1 mediated rapid protection against bubonic plague. Vaccine.

[34] Levy Y., Vagima Y., Tidhar A., Aftalion M., Gur D., Nili U., Chitlaru T., Zauberman A., Mamroud E. (2018). Targeting of the Yersinia pestis F1 capsular antigen by innate-like B1b cells mediates a rapid protective response against bubonic plague. NPJ Vaccines.

[35] Aftalion M., Tidhar A., Vagima Y., Gur D., Zauberman A., Holtzman T., Makovitzki A., Chitlaru T., Mamroud E., Levy Y. (2023). Rapid Induction of Protective Immunity against Pneumonic Plague by Yersinia pestis Polymeric F1 and LcrV Antigens. Vaccines.

[36] Gu H., Zeng X., Peng L., Xiang C., Zhou Y., Zhang X., Zhang J., Wang N., Guo G., Li Y. (2021). Vaccination induces rapid protection against bacterial pneumonia via training alveolar macrophage in mice. Elife.

[37] Nathan C. (2006). Neutrophils and immunity: Challenges and opportunities. Nat. Rev. Immunol..

[38] Rosales C. (2018). Neutrophil: A Cell with Many Roles in Inflammation or Several Cell Types?. Front. Physiol..

[39] Murphy J., Summer R., Wilson A.A., Kotton D.N., Fine A. (2008). The prolonged life-span of alveolar macrophages. Am. J. Respir. Cell. Mol. Biol..

[40] Viola M.F., Boeckxstaens G. (2020). Intestinal resident macrophages: Multitaskers of the gut. Neurogastroenterol. Motil..

[41] Patel A.A., Ginhoux F., Yona S. (2021). Monocytes, macrophages, dendritic cells and neutrophils: An update on lifespan kinetics in health and disease. Immunology.

[42] Pillay J., den Braber I., Vrisekoop N., Kwast L.M., de Boer R.J., Borghans J.A., Tesselaar K., Koenderman L. (2010). In vivo labeling with 2H_2_O reveals a human neutrophil lifespan of 5.4 days. Blood.

[43] Koenderman L., Tesselaar K., Vrisekoop N. (2022). Human neutrophil kinetics: A call to revisit old evidence. Trends Immunol..

[44] Segel G.B., Halterman M.W., Lichtman M.A. (2011). The paradox of the neutrophil’s role in tissue injury. J. Leukoc. Biol..

[45] Kruger P., Saffarzadeh M., Weber A.N., Rieber N., Radsak M., von Bernuth H., Benarafa C., Roos D., Skokowa J., Hartl D. (2015). Neutrophils: Between host defence, immune modulation, and tissue injury. PLoS Pathog..

[46] Eichelberger K.R., Jones G.S., Goldman W.E. (2019). Inhibition of Neutrophil Primary Granule Release during Yersinia pestis Pulmonary Infection. mBio.

[47] Nasser A., Moradi M., Jazireian P., Safari H., Alizadeh-Sani M., Pourmand M.R., Azimi T. (2019). *Staphylococcus aureus* versus neutrophil: Scrutiny of ancient combat. Microb. Pathog..

[48] Dahlgren C., Gabl M., Holdfeldt A., Winther M., Forsman H. (2016). Basic characteristics of the neutrophil receptors that recognize formylated peptides, a danger-associated molecular pattern generated by bacteria and mitochondria. Biochem. Pharmacol..

[49] Wen X., Xu X., Sun W., Chen K., Pan M., Wang J.M., Bolland S.M., Jin T. (2019). G-protein-coupled formyl peptide receptors play a dual role in neutrophil chemotaxis and bacterial phagocytosis. Mol. Biol. Cell.

[50] Liu M., Chen K., Yoshimura T., Liu Y., Gong W., Wang A., Gao J.L., Murphy P.M., Wang J.M. (2012). Formylpeptide receptors are critical for rapid neutrophil mobilization in host defense against Listeria monocytogenes. Sci. Rep..

[51] Oldekamp S., Pscheidl S., Kress E., Soehnlein O., Jansen S., Pufe T., Wang J.M., Tauber S.C., Brandenburg L.O. (2014). Lack of formyl peptide receptor 1 and 2 leads to more severe inflammation and higher mortality in mice with of pneumococcal meningitis. Immunology.

[52] Weiss E., Schlatterer K., Beck C., Peschel A., Kretschmer D. (2020). Formyl-Peptide Receptor Activation Enhances Phagocytosis of Community-Acquired Methicillin-Resistant Staphylococcus aureus. J. Infect. Dis..

[53] Jeong Y.S., Bae Y.S. (2020). Formyl peptide receptors in the mucosal immune system. Exp. Mol. Med..

[54] Showell H.J., Freer R.J., Zigmond S.H., Schiffmann E., Aswanikumar S., Corcoran B., Becker E.L. (1976). The structure-activity relations of synthetic peptides as chemotactic factors and inducers of lysosomal secretion for neutrophils. J. Exp. Med..

[55] Tsai Y.F., Yang S.C., Hwang T.L. (2016). Formyl peptide receptor modulators: A patent review and potential applications for inflammatory diseases (2012–2015). Expert Opin. Ther. Pat..

[56] Zhuang Y., Wang L., Guo J., Sun D., Wang Y., Liu W., Xu H.E., Zhang C. (2022). Molecular recognition of formylpeptides and diverse agonists by the formylpeptide receptors FPR1 and FPR2. Nat. Commun..

[57] Schepetkin I.A., Khlebnikov A.I., Giovannoni M.P., Kirpotina L.N., Cilibrizzi A., Quinn M.T. (2014). Development of small molecule non-peptide formyl peptide receptor (FPR) ligands and molecular modeling of their recognition. Curr. Med. Chem..

[58] Lind S., Dahlgren C., Holmdahl R., Olofsson P., Forsman H. (2021). Functional selective FPR1 signaling in favor of an activation of the neutrophil superoxide generating NOX2 complex. J. Leukoc. Biol..

[59] Nanamori M., Cheng X., Mei J., Sang H., Xuan Y., Zhou C., Wang M.W., Ye R.D. (2004). A novel nonpeptide ligand for formyl peptide receptor-like 1. Mol. Pharmacol..

[60] Reshetnikov V., Hahn J., Maueroder C., Czegley C., Munoz L.E., Herrmann M., Hoffmann M.H., Mokhir A. (2018). Chemical Tools for Targeted Amplification of Reactive Oxygen Species in Neutrophils. Front. Immunol..

[61] Payne J.A.E., Tailhades J., Ellett F., Kostoulias X., Fulcher A.J., Fu T., Leung R., Louch S., Tran A., Weber S.A. (2021). Antibiotic-chemoattractants enhance neutrophil clearance of Staphylococcus aureus. Nat. Commun..

[62] Brinkmann V., Reichard U., Goosmann C., Fauler B., Uhlemann Y., Weiss D.S., Weinrauch Y., Zychlinsky A. (2004). Neutrophil extracellular traps kill bacteria. Science.

[63] Monteith A.J., Miller J.M., Maxwell C.N., Chazin W.J., Skaar E.P. (2021). Neutrophil extracellular traps enhance macrophage killing of bacterial pathogens. Sci. Adv..

[64] Remijsen Q., Kuijpers T.W., Wirawan E., Lippens S., Vandenabeele P., Vanden Berghe T. (2011). Dying for a cause: NETosis, mechanisms behind an antimicrobial cell death modality. Cell Death Differ..

[65] Peng L., Li L., He X.L., Yu J.Y., Zeng Z.J., Yang W.J., Zhang B., Zhang T.S., Cao H., Huang S.H. (2020). Memantine Displays Antimicrobial Activity by Enhancing Escherichia coli Pathogen-Induced Formation of Neutrophil Extracellular Traps. Front. Cell. Infect. Microbiol..

[66] Xie T., Duan Z., Sun S., Chu C., Ding W. (2021). beta-Lactams modulate neutrophil extracellular traps formation mediated by mTOR signaling pathway. Biochem. Biophys. Res. Commun..

[67] Chiang N., Sakuma M., Rodriguez A.R., Spur B.W., Irimia D., Serhan C.N. (2022). Resolvin T-series reduce neutrophil extracellular traps. Blood.

[68] Fisher J., Mohanty T., Karlsson C.A.Q., Khademi S.M.H., Malmstrom E., Frigyesi A., Nordenfelt P., Malmstrom J., Linder A. (2021). Proteome Profiling of Recombinant DNase Therapy in Reducing NETs and Aiding Recovery in COVID-19 Patients. Mol. Cell. Proteom..

[69] Irizarry-Caro J.A., Carmona-Rivera C., Schwartz D.M., Khaznadar S.S., Kaplan M.J., Grayson P.C. (2018). Brief Report: Drugs Implicated in Systemic Autoimmunity Modulate Neutrophil Extracellular Trap Formation. Arthritis Rheumatol..

[70] Borchers A.T., Keen C.L., Gershwin M.E. (2007). Drug-induced lupus. Ann. N. Y. Acad. Sci..

[71] Xu L., Zhang W., Kwak M., Zhang L., Lee P.C.W., Jin J.O. (2019). Protective Effect of Melatonin Against Polymicrobial Sepsis Is Mediated by the Anti-bacterial Effect of Neutrophils. Front. Immunol..

[72] Song Y., Kadiyala U., Weerappuli P., Valdez J.J., Yalavarthi S., Louttit C., Knight J.S., Moon J.J., Weiss D.S., VanEpps J.S. (2019). Antimicrobial Microwebs of DNA-Histone Inspired from Neutrophil Extracellular Traps. Adv. Mater..

[73] Chen Y.F., Chiou Y.H., Chen Y.C., Jiang Y.S., Lee T.Y., Jan J.S. (2021). ZnO-loaded DNA nanogels as neutrophil extracellular trap-like structures in the treatment of mouse peritonitis. Mater. Sci. Eng. C Mater. Biol. Appl..

[74] Tram N.D.T., Xu J., Mukherjee D., Obanel A.E., Mayandi V., Selvarajan V., Zhu X., Teo J., Barathi V.A., Lakshminarayanan R. (2022). Bacteria-Responsive Self-Assembly of Antimicrobial Peptide Nanonets for Trap-and-Kill of Antibiotic-Resistant Strains. Adv. Funct. Mater..

[75] Huang Z., Liu Y., Wang L., Ali A., Yao Q., Jiang X., Gao Y. (2020). Supramolecular assemblies mimicking neutrophil extracellular traps for MRSE infection control. Biomaterials.

[76] Antelo G.T., Vila A.J., Giedroc D.P., Capdevila D.A. (2021). Molecular Evolution of Transition Metal Bioavailability at the Host-Pathogen Interface. Trends Microbiol..

[77] Murdoch C.C., Skaar E.P. (2022). Nutritional immunity: The battle for nutrient metals at the host-pathogen interface. Nat. Rev. Microbiol..

[78] Gomes A.C., Moreira A.C., Mesquita G., Gomes M.S. (2018). Modulation of Iron Metabolism in Response to Infection: Twists for All Tastes. Pharmaceuticals.

[79] Sheldon J.R., Laakso H.A., Heinrichs D.E. (2016). Iron Acquisition Strategies of Bacterial Pathogens. Microbiol. Spectr..

[80] Krause A., Neitz S., Magert H.J., Schulz A., Forssmann W.G., Schulz-Knappe P., Adermann K. (2000). LEAP-1, a novel highly disulfide-bonded human peptide, exhibits antimicrobial activity. FEBS Lett..

[81] Park C.H., Valore E.V., Waring A.J., Ganz T. (2001). Hepcidin, a urinary antimicrobial peptide synthesized in the liver. J. Biol. Chem..

[82] Ganz T. (2011). Hepcidin and iron regulation, 10 years later. Blood.

[83] Moreira A.C., Neves J.V., Silva T., Oliveira P., Gomes M.S., Rodrigues P.N. (2017). Hepcidin-(In)dependent Mechanisms of Iron Metabolism Regulation during Infection by Listeria and Salmonella. Infect. Immun..

[84] Peyssonnaux C., Zinkernagel A.S., Datta V., Lauth X., Johnson R.S., Nizet V. (2006). TLR4-dependent hepcidin expression by myeloid cells in response to bacterial pathogens. Blood.

[85] Arezes J., Jung G., Gabayan V., Valore E., Ruchala P., Gulig P.A., Ganz T., Nemeth E., Bulut Y. (2015). Hepcidin-induced hypoferremia is a critical host defense mechanism against the siderophilic bacterium Vibrio vulnificus. Cell Host Microbe.

[86] Armitage A.E., Eddowes L.A., Gileadi U., Cole S., Spottiswoode N., Selvakumar T.A., Ho L.P., Townsend A.R., Drakesmith H. (2011). Hepcidin regulation by innate immune and infectious stimuli. Blood.

[87] Wessling-Resnick M. (2015). Nramp1 and Other Transporters Involved in Metal Withholding during Infection. J. Biol. Chem..

[88] Tolosano E., Altruda F. (2002). Hemopexin: Structure, function, and regulation. DNA Cell Biol..

[89] Wagener B.M., Hu P.J., Oh J.Y., Evans C.A., Richter J.R., Honavar J., Brandon A.P., Creighton J., Stephens S.W., Morgan C. (2019). Correction: Role of heme in lung bacterial infection after trauma hemorrhage and stored red blood cell transfusion: A preclinical experimental study. PLoS Med..

[90] Sakamoto K., Kim Y.G., Hara H., Kamada N., Caballero-Flores G., Tolosano E., Soares M.P., Puente J.L., Inohara N., Nunez G. (2017). IL-22 Controls Iron-Dependent Nutritional Immunity Against Systemic Bacterial Infections. Sci. Immunol..

[91] Weiss G. (2023). Macrophage vesicles starve bacteria of iron. Nat. Metab..

[92] Kuang H., Dou G., Cheng L., Wang X., Xu H., Liu X., Ding F., Yang X., Liu S., Bao L. (2023). Humoral regulation of iron metabolism by extracellular vesicles drives antibacterial response. Nat. Metab..

[93] Lonnerdal B., Iyer S. (1995). Lactoferrin: Molecular structure and biological function. Annu. Rev. Nutr..

[94] Corbin B.D., Seeley E.H., Raab A., Feldmann J., Miller M.R., Torres V.J., Anderson K.L., Dattilo B.M., Dunman P.M., Gerads R. (2008). Metal chelation and inhibition of bacterial growth in tissue abscesses. Science.

[95] Zygiel E.M., Nolan E.M. (2018). Transition Metal Sequestration by the Host-Defense Protein Calprotectin. Annu. Rev. Biochem..

[96] Goetz D.H., Holmes M.A., Borregaard N., Bluhm M.E., Raymond K.N., Strong R.K. (2002). The neutrophil lipocalin NGAL is a bacteriostatic agent that interferes with siderophore-mediated iron acquisition. Mol. Cell.

[97] Flo T.H., Smith K.D., Sato S., Rodriguez D.J., Holmes M.A., Strong R.K., Akira S., Aderem A. (2004). Lipocalin 2 mediates an innate immune response to bacterial infection by sequestrating iron. Nature.

[98] Holmes M., Paulsene W., Jide X., Ratledge C., Strong R.K. (2005). Siderocalin (Lcn2) also binds carboxymycobactins, potentially defending against Mycobacterial infections through iron sequestration. Structure.

[99] Sheldon J.R., Himmel L.E., Kunkle D.E., Monteith A.J., Maloney K.N., Skaar E.P. (2022). Lipocalin-2 is an essential component of the innate immune response to Acinetobacter baumannii infection. PLoS Pathog..

[100] Islam S., Jarosch S., Zhou J., Parquet Mdel C., Toguri J.T., Colp P., Holbein B.E., Lehmann C. (2016). Anti-inflammatory and anti-bacterial effects of iron chelation in experimental sepsis. J. Surg. Res..

[101] Fernandes S.S., Nunes A., Gomes A.R., de Castro B., Hider R.C., Rangel M., Appelberg R., Gomes M.S. (2010). Identification of a new hexadentate iron chelator capable of restricting the intramacrophagic growth of Mycobacterium avium. Microbes Infect..

[102] Bakhshandeh Z., Halabian R., Imani Fooladi A.A., Jahanian-Najafabadi A., Jalili M.A., Roudkenar M.H. (2014). Recombinant human lipocalin 2 acts as an antibacterial agent to prevent platelet contamination. Hematology.

[103] Bento C.M., Gomes M.S., Silva T. (2020). Looking beyond Typical Treatments for Atypical Mycobacteria. Antibiotics.

[104] Savage K.A., Parquet M.C., Allan D.S., Davidson R.J., Holbein B.E., Lilly E.A., Fidel P.L. (2018). Iron Restriction to Clinical Isolates of Candida albicans by the Novel Chelator DIBI Inhibits Growth and Increases Sensitivity to Azoles In Vitro and In Vivo in a Murine Model of Experimental Vaginitis. Antimicrob. Agents Chemother..

[105] Chawla L.S., Beers-Mulroy B., Tidmarsh G.F. (2019). Therapeutic Opportunities for Hepcidin in Acute Care Medicine. Crit. Care Clin..

[106] Xiao X., Yeoh B.S., Vijay-Kumar M. (2017). Lipocalin 2: An Emerging Player in Iron Homeostasis and Inflammation. Annu. Rev. Nutr..

[107] Asaf S., Maqsood F., Jalil J., Sarfraz Z., Sarfraz A., Mustafa S., Ojeda I.C. (2022). Lipocalin 2-not only a biomarker: A study of current literature and systematic findings of ongoing clinical trials. Immunol. Res..

[108] Wang C.H., Hsieh Y.H., Powers Z.M., Kao C.Y. (2020). Defeating Antibiotic-Resistant Bacteria: Exploring Alternative Therapies for a Post-Antibiotic Era. Int. J. Mol. Sci..

